# An Unusual Presentation of Peri‐Lead Edema Following Deep Brain Stimulation for Parkinson's Disease: A Case Report and Review of the Literature

**DOI:** 10.1002/ccr3.70221

**Published:** 2025-03-06

**Authors:** V. S. Witzig, R. Pjontek, A. Höllig, J. Schiefer, M. Wiesmann, H. Clusmann, J. B. Schulz, F. Holtbernd

**Affiliations:** ^1^ Department of Neurology RWTH Aachen University Aachen Germany; ^2^ Department of Neurosurgery RWTH Aachen University Aachen Germany; ^3^ Department of Stereotactic and Functional Neurosurgery Faculty of Medicine, University Hospital Cologne, University of Cologne Cologne Germany; ^4^ Department of Neuroradiology University Hospital RWTH Aachen Aachen Germany; ^5^ JARA‐BRAIN Institute Molecular Neuroscience and Neuroimaging Juelich Research Center GmbH and RWTH Aachen University Aachen Germany; ^6^ Juelich Research Center Institutes of Neuroscience and Medicine (INM‐4, INM‐11) Juelich Germany

**Keywords:** deep brain stimulation, movement disorders, neuromodulation, Parkinson's disease, peri‐lead edema

## Abstract

Peri‐lead edema (PLE) after deep brain stimulation may mimic brain infection on magnetic resonance imaging (MRI). We present a case of symptomatic PLE with annular contrast enhancement on MRI suggestive of an infectious cause. We show that careful clinical evaluation and laboratory testing, in addition to neuroimaging, are essential to guide treatment and to avoid unnecessary interventions in PLE cases with a favorable spontaneous course.

## 1 Introduction

Deep brain stimulation (DBS) is an effective treatment for movement disorders [1]. Whereas relevant tissue damage caused by electrode placement is rarely observed after DBS surgery [2], transient brain edema along the electrode's trajectory (peri‐lead edema [PLE]) is not uncommon [3]. However, the reported frequency and time of occurrence vary substantially across studies [3, 4, 5, 6, 7, 8], and the underlying pathophysiology remains elusive [9, 10, 11]. Most patients developing PLE are asymptomatic, which likely results in an underestimation of the true occurrence [3, 5, 7, 12]. The clinical presentation of symptomatic PLE is heterogeneous and includes confusion, altered consciousness, focal neurological deficits, and seizures [13]. PLE is diagnosed using brain computed tomography (CT) or magnetic resonance imaging (MRI) [3, 4, 7, 10, 14]. CT typically reveals hypodensities around the electrode [15]. MRI shows a T2 hyperintense signal of variable spatial extent along the electrode. Alterations on diffusion‐weighted imaging (DWI) and hemorrhage may be present [3, 4, 5, 11], but in most cases, susceptibility weighted imaging does not provide evidence of bleeding [3, 4, 8, 10]. PLE‐associated contrast enhancement (CE) on MRI is very rare, and annular CE is a typical feature of brain abscess formation rather than edema [4, 16].

Here, we report an unusual presentation of symptomatic PLE with marked annular CE on MRI, suggestive of a brain abscess with a favorable clinical outcome without electrode removal.

## 2 Case History/Examination

**FIGURE 1:**
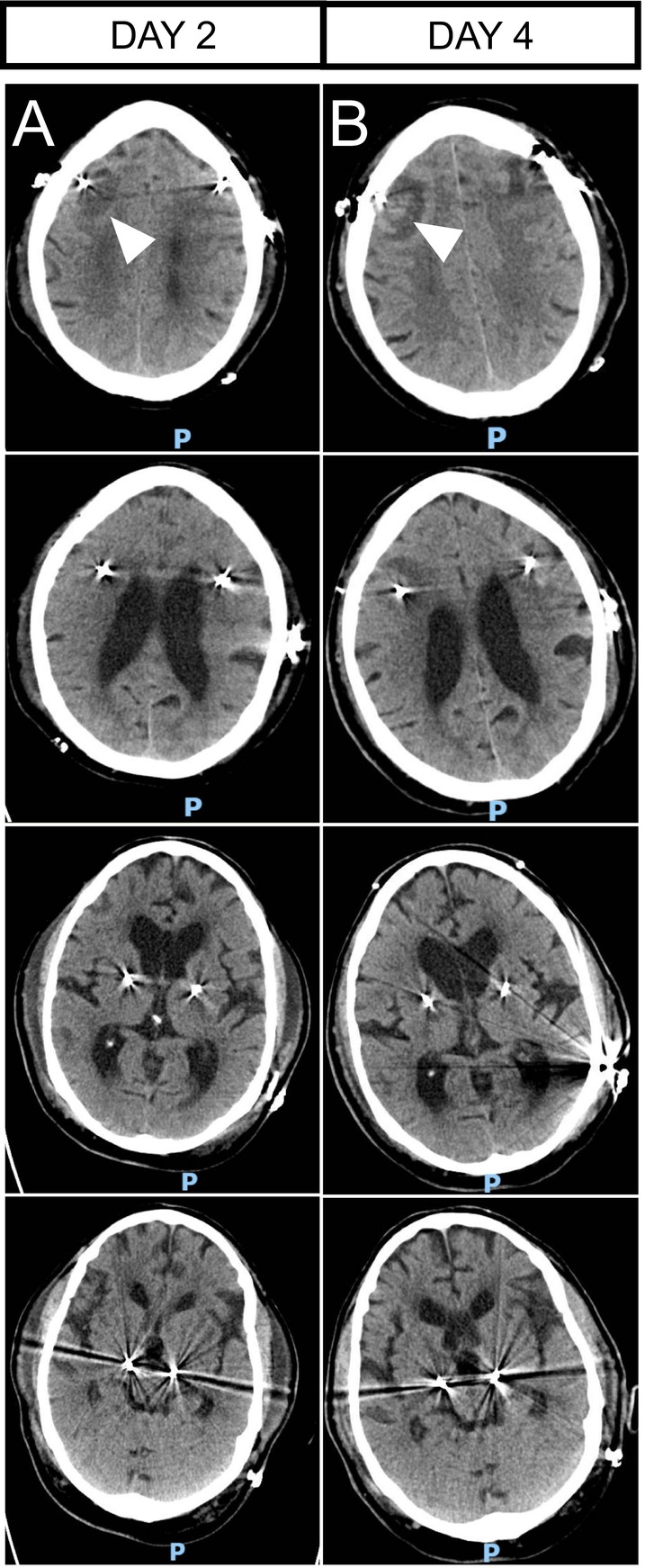
Exemplary radiological findings of longitudinal CT scans. (A) CT on postoperative day two showed bifrontal hypodensities around the electrodes. (B) CT on postoperative day 4 revealed progressive edema (marked with arrows).

**FIGURE 2:**
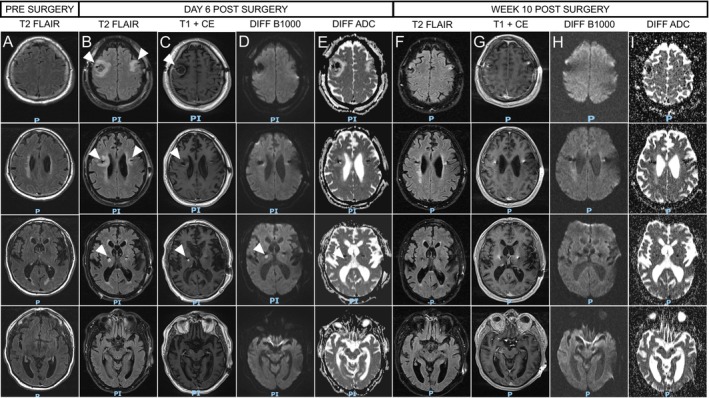
Longitudinal evaluation of brain MRI. (A) MRI pre surgery showing several microvascular white matter lesions. (B) Extensive edema as reflected by hyperintensities on T2‐weighted fluid‐attenuated inversion recovery (FLAIR) MRI on day 6 post‐surgery along the electrodes' trajectories, pronounced on the right side (marked with arrows). (C) Bilateral cortical annular contrast enhancement (CE) on both sides, marked on the right side (marked with arrows). (D) Diffusion weighted imaging (DWI) revealed no signal restriction in the area of annular CE (limited accessibility due to metal artifacts) but suggested right thalamic infarction (marked with arrow) with corresponding signal on the ADC‐map (E). (F) Follow‐up MRI at week 10 postsurgery showed near complete remission of edema on FLAIR imaging with complete remission of CE on T1‐weighted MRI (G). There was no sign of (chronic) ischemic infarction in the right thalamus (H, I).

A 71‐year‐old male, Caucasian patient with the monogenetic form of Parkinson's disease (PD), caused by a heterozygote mutation in the leucine‐rich‐repeat‐kinase‐2 (LRRK‐2) gene with severe motor fluctuations (Hoehn and Yahr Stage III, off medication motor unified PD rating scale [UPDRS] part III 51 points, levodopa equivalent daily dose [LEDD 1246 mg]), underwent bilateral implantation of electrodes in the subthalamic nucleus (STN) for DBS (device: Medtronic Percept PC with SenSight directional leads). The surgery was done asleep and lasted for approximately 5 h. The routine postoperative CT scan (on the same day) was unremarkable other than mild pneumocephalus, with correct localization of both electrodes. On the first day after surgery, the patient developed a severe hyperactive delirium, which required symptomatic therapy with quetiapine. On postoperative day 2, delirium improved, but the patient was still not fully oriented, and consciousness was reduced. CT on Day 2 revealed bifrontal hypodensities along the electrodes (Figure 1A). The clinical status of the patient further improved, and the delirious state completely resolved during the following days. That said, CT on Day 4 revealed progressive edema (Figure 1B), which is why an MRI with contrast was performed on Day 6. Preoperative MRI had been unremarkable except for microvascular lesions (Figure 2A). MRI showed extensive T2 hyperintensities along the electrodes' trajectories, pronounced on the right side, expanding from the cortex to the deep white matter and thalamus (Figure 2B). Susceptibility weighted imaging suggested the presence of cortical hemorrhage in the proximity of the electrodes' insertion site on the right side. There was bilateral cortical annular CE on both sides, marked on the right side, suggestive of an underlying infectious cause (Figure 2C). DWI revealed a hyperintense signal in the right thalamus adjacent to the electrode, suggesting brain infarction. We did not observe hyperintensities in the region adjacent to the electrodes presenting with annular CE on DWI. However, due to metal‐induced artifacts, this region could not be assessed reliably (Figure 2D). The apparent diffusion coefficient (ADC) map showed an increased signal (Figure 2E), which is not typically observed in brain abscesses and thus can be interpreted as a shine‐through effect. Contrasting the MRI findings, the patient did not show clinical signs of infection or thalamic infarction, and a typical microlesion effect was observed, resulting in a marked reduction of hypokinesia and rigidity. Tests for infection showed mild elevation of C‐reactive protein, which decreased from 127 mg/dL (Day 3) to 13 mg/dL on Day 15 (reference < 5 mg/dL) with normal white blood cell counts. Blood cultures and analysis of cerebrospinal fluid were unrevealing. Thus, an infectious cause was regarded unlikely, and the decision was made to not administer antibiotic therapy or remove the electrodes. DBS was initiated on the fifth postoperative day, and the patient responded well. The patient was discharged 19 days after surgery.

## 3 Results (Outcome and Follow‐Up)

On clinical follow‐up 10 weeks after surgery, an MRI was repeated, showing near‐complete remission of edema (Figure 2F). There was no CE (Figure 2G), and susceptibility weighted imaging was not suggestive of hemorrhage. T2‐weighted imaging did not reveal signs of gliosis in the right thalamus, where brain ischemia was suspected on initial MRI. DWI at follow‐up did not reveal any signs of brain ischemia (Figure 2H,I). On continued clinical follow‐up over 10 months, the patient showed sustained responsiveness to DBS, motor fluctuations were substantially alleviated, and the LEDD was reduced by 476 mg.

## 4 Discussion

### 4.1 Peri‐Lead Edema

#### 4.1.1 Epidemiology

PLE was first described in 2004 in a retrospective analysis of 38 patients receiving DBS for STN (*n* = 31), globus pallidus internus (*n* = 2), and nucleus ventralis intermedius of the thalamus (*n* = 5). In all, 39% of patients showed signs of PLE on MRI [7]. All patients were asymptomatic. A limitation of this study was the rather long latency between surgery and the time of imaging, ranging from 1 to more than 6 months [7]. Initially, PLE was regarded as a rare complication of DBS, but more recent studies suggest that PLE is much more common. Indeed, in the only available prospective study, including 19 patients with PD who underwent MRI between 7 and 20 days after surgery, all subjects showed PLE of variable degree. Unilateral hemorrhage along the electrode was present in four patients [3]. In none of the cases, CE was detected. Importantly, however, the application of the contrast agent was discontinued after the first 3 patients because of the negative findings. A total of 31.6% of PLE cases were symptomatic. There was no correlation between the extent of edema and the presence of clinical symptoms [3]. A recently published systematic review [11], including 10 studies, reported a PLE frequency of 35.8%, of which 3.1% were symptomatic. Although the time of appearance is heterogeneous, PLE appears to develop around Day 4 after surgery in most cases. That being said, neuroimaging may remain positive for up to 3 months [3, 14]. Even though there are no established risk factors for the development of PLE [17], the presence of gray matter atrophy may be associated with an increased risk of PLE [18].

The high variability of the reported occurrence can be partially explained by differences in the imaging modality applied (CT vs. MRI) and the different time points at which imaging was conducted. As PLE is asymptomatic in most cases and hardly ever develops within the first 24 h after surgery [6, 8] when postoperative CT scanning is routinely performed, PLE may often remain undetected [3].

#### 4.1.2 Pathophysiology

The pathophysiology underlying PLE is unknown. Most likely, PLE is a vasogenic edema caused by mechanical irritation, leading to CSF accumulation and disruption of the blood–brain barrier along the electrode's trajectory. Immune processes induced by the implant material may also be involved, but there is little evidence for toxic processes [9, 11]. Increased blood–brain barrier permeability facilitates the invasion of inflammatory cells and release of cytokines [11]. The possibility of ‘CSF tracking’ along the electrode's trajectory has also been discussed as a cause of PLE [9]. However, the trajectory did not pass the lateral ventricles in our case, deeming this possibility unlikely. Venous infarction caused by injury and obliteration of cortical veins during the opening of the dura may also contribute to PLE [11]. However, extensive subcortical edema can hardly be explained by the occlusion of single superficial veins. It has also been suggested that directional leads may increase the risk of PLE due to more severe mechanical irritation from electrode rotation and different surface characteristics [19]. Interestingly, a recent study has reported an association of gray matter atrophy and PLE occurrence in PD [18]. This association may explain why the majority of PLE cases have been reported in PD. DBS in PD patients is rarely performed prior to 5 years of disease duration. Moreover, brain atrophy is common in the course of PD [20]. Thus, the magnitude of brain atrophy is expected to be more severe in most PD cases admitted to DBS compared with that in dystonia or ET patients. Along these lines, to our knowledge, PLE has never been reported following lead implantation for stereoelectroencephalography in epilepsy patients. Alternatively, the seemingly higher rate of PLE in PD may simply reflect the larger number of surgeries performed on these patients.

#### 4.1.3 Clinical Symptoms

Most patients with PLE are asymptomatic [3, 5, 7, 12]. Moreover, some symptoms in the course of DBS surgery may not be associated with PLE but rather may be caused by postoperative delirium, no matter which reason surgery was done [21, 22]. If PLE is symptomatic, there is a wide variability of symptom severity. Clinical presentation ranges from mild confusion and behavioral changes to lethargy, severe hypokinesia, seizures, and focal neurological deficits such as cranial nerve deficiencies, diplopia, aphasia, and hemiparesis [13, 23, 24]. Epileptic seizures appear to be more prevalent in patients with subcortical edema [8]. Clinical symptoms usually emerge around 4 days after surgery and resolve after a few weeks, lasting up to 3 months in some cases [3, 6, 8]. Both immediate onset (< 24 h) and long‐lasting symptoms are exceptions [6, 8, 17, 23]. Of note, neuroimaging may reveal signs of PLE long after clinical symptoms have ceased [3, 17, 25]. Interestingly, even though edema forms bilaterally in most instances, patients usually present with unilateral deficits [5, 8, 9, 11, 17, 23, 25, 26]. Life‐threatening PLE symptoms are rare. In one report, the patient showed acute onset, prolonged severe reduction of consciousness, aphasia, and hemiparesis following thalamic DBS for a mixed tremor [23]. Reduced consciousness necessitated intubation and long‐term treatment in an intensive care unit, including tracheostomy and tube feeding [23].

#### 4.1.4 Diagnosis and Therapy

There are no standard criteria for diagnosing PLE. CT scans show low‐attenuation signal with or without diffuse hemorrhage and may present cystic cavitation [10]. MRI typically reveals a T2‐hyperintense signal extending circumferentially along the electrode, best observed on fluid‐attenuated inversion recovery imaging, reflecting edema. In contrast, T1‐weighted imaging reveals a hypointense signal. Microbleeds are rare; DWI may suggest ischemia [3, 7, 11, 14, 24]. That said, follow‐up imaging usually does not show permanent tissue damage, speaking against the occurrence of brain infarction in the course of PLE [24, 27]. To our knowledge, to date, only five cases with PLE associated with CE on MRI have been reported [4, 14, 28]. If CE is present, PLE is difficult to distinguish from brain abscess. Electrode‐related brain abscess is a rare but well‐known complication of DBS surgery [29, 30]. If CE is caused by brain abscess, patients typically show systemic signs of infection [16, 31]. Moreover, the development of abscess formation usually does not emerge during the first days after surgery [16, 31]. Due to artifacts caused by the lead implants, the interpretation of DWI may be difficult. However, similar to our report, Innocenti et al. did observe an increased ADC signal in the alleged brain abscess cavity [28], which is not typical of brain abscess formation [32] and may aid in differential diagnosis.

There is no evidence‐based treatment for PLE. Because of the assumed vasogenic etiology, some authors have suggested the administration of dexamethasone [13], but prospective studies have not confirmed a positive effect [3]. Given the self‐limited nature of PLE, specific treatment may not be required in asymptomatic or mildly affected patients, and symptom‐specific treatment of seizures and delirium may be sufficient. However, in cases with severe symptoms and rapid progression, corticosteroids may be a valid option [23].

DBS is evolving and its spectrum of application is expanding continuously. Thus, it is important to recognize and understand its potential complications. Whereas there is general awareness of DBS surgery‐related stroke, intracranial hemorrhage, implant‐associated infections, and material failure [4, 13, 17, 25, 33], PLE may not be appreciated accordingly. Recently published work suggests that PLE is much more common than initially assumed [3]. As most cases remain asymptomatic, PLE likely is underdiagnosed. Even though no specific treatment may be needed in most cases, it is important for neurosurgeons, neurologists, and neuroradiologists to know about the heterogeneous clinical and imaging presentations to avoid unnecessary antibiotic therapy or even electrode removal. Our case confirms that CE on MRI can occur in PLE and does not necessarily hint toward an infectious cause. Furthermore, our case emphasizes that the clinical course and results of laboratory workup rather than imaging findings should direct the treatment strategy. The clinical status of our patient improved quickly and spontaneously without the application of dexamethasone. It is possible that symptoms were not uniquely related to PLE but that the short‐lasting delirium was associated with the surgery and general anesthesia per se. In line with previous reports [13, 23, 24], we found mild signs of PLE on MRI follow‐up, long after symptoms had subsided. MRI follow‐up suggested that increased diffusion in the thalamus on initial MRI was not caused by brain infarction [24, 27].

## 5 Conclusion

We present an unusual case of PLE following DBS for PD, presenting with marked CE on MRI and a favorable clinical outcome. Our case illustrates that the presence of CE is not unequivocally associated with implant‐related infection but can be a feature of PLE. We suggest that the clinical course, rather than imaging findings, should guide the decision of electrode removal.

## Author Contributions

**V. S. Witzig:** conceptualization, data curation, investigation, methodology, project administration, validation, visualization, writing – original draft, writing – review and editing. **R. Pjontek:** investigation, writing – review and editing. **A. Höllig:** investigation, writing – review and editing. **J. Schiefer:** investigation, writing – review and editing. **M. Wiesmann:** investigation, writing – review and editing. **H. Clusmann:** investigation, writing – review and editing. **J. B. Schulz:** resources, writing – review and editing. **F. Holtbernd:** conceptualization, project administration, supervision, writing – original draft, writing – review and editing.

## Ethics Statement

The authors have nothing to report.

## Consent

Written informed consent was obtained from the patient for the publication of the details of his medical case and any accompanying images.

## Conflicts of Interest

M.W. is working as a consultant for Stryker Neurovascular. M.W. has been working as a consultant for Kaneka Pharmaceuticals and Medtronic (both inactive). M.W. has received reimbursement for lectures or travel support from Bracco Imaging, Medtronic, Siemens Healthcare, and Stryker Neurovascular. M.W. has received grants for research projects or educational exhibits from ab medica, Acandis, Bayer, Bracco Imaging, Cerenovus, Codman Neurovascular, Dahlhausen, Kaneka Pharmaceuticals, Medtronic, Mentice AB, Microvention, Phenox, Siemens Healthcare, and Stryker Neurovascular. J.B.S. is working in the advisory board of Forward Pharma, MSD, Lundbeck, Biogen, Eisai, Novo Nordisk, Roche, Reata, and Lilly. J.B.S. has received reimbursement for lectures from Merz, Teva, Bayer, UCB, Lilly, Boehringer, GSK, Bial, Novartis, Biogen, and Eisai. J.B.S. has received grants for research projects from Biogen, Eisai, and Lilly. F.H. received travel and conference fees from Bial, Desitin, Abbott, Zambon, and Abbvie. V.S.W. received travel and conference fees from Medtronic. The other authors declare no conflicts of interest.

## Data Availability

Data sharing is not applicable to this article as no datasets were generated or analyzed during the current study.
